# Innovationsfonds und Primärversorgung – Welche Erwartungen und Erfahrungen vertreten Hausärzt*innen in Bezug auf die Teilnahme an innovativen Versorgungsmodellen?

**DOI:** 10.1007/s00103-022-03533-y

**Published:** 2022-04-27

**Authors:** Julian Wangler, Michael Jansky

**Affiliations:** Zentrum für Allgemeinmedizin und Geriatrie, Universitätsmedizin Mainz, Am Pulverturm 13, 55131 Mainz, Deutschland

**Keywords:** Innovationsfonds, Versorgungsforschung, Hausarzt, Primärversorgung, Rekrutierung, Innovation Fund, Health services research, General practitioner, Primary care, Recruitment

## Abstract

**Hintergrund:**

Zur Verbesserung der medizinischen Versorgung wurde im Jahr 2015 der Innovationsfonds eingerichtet. Damit Interventionen bzw. neue Versorgungsformen erprobt und perspektivisch in die Regelversorgung übernommen werden können, bedarf es der Einbeziehung der allgemeinmedizinischen Versorgung.

**Ziel der Arbeit:**

Die Studie exploriert hausärztliche Einstellungen, teilnahmerelevante Erwartungen und Erfahrungen mit Blick auf Innovationsfondsprojekte.

**Methoden:**

Zwischen Juli und Oktober 2021 wurden sämtliche 13.170 als Behandler*innen aktive Hausärzt*innen in Baden-Württemberg, Hessen und Rheinland-Pfalz zu einer Onlinebefragung eingeladen. 3556 vollständig ausgefüllte Fragebögen gingen in die Auswertung ein (Rücklauf: 27 %). Neben der deskriptiven Analyse kam zur Feststellung von signifikanten Unterschieden zwischen 2 Gruppen ein *t*-Test bei unabhängigen Stichproben zum Einsatz.

**Ergebnisse:**

83 % der Befragten kennen den Innovationsfonds. Die Befragten verbinden ihn mehrheitlich mit Chancen und Potenzialen (u. a. Intensivierung anwendungsnaher Versorgungsforschung, unabhängige Finanzierung, Einbeziehung der Primärversorgung). Dennoch sind sich viele Hausärzt*innen unsicher, inwiefern speziell die Primärversorgung längerfristig vom Innovationsfonds wird profitieren können. Hinsichtlich der Bereitschaft zur Mitwirkung an Innovationsfondsstudien zeigen sich die Befragten gespalten. Befragte, die bereits an Innovationsfondsprojekten teilgenommen haben (24 %), ziehen eine überwiegend positive Bilanz (Nutzen der Intervention, Aufwand-Nutzen-Verhältnis). Dennoch werden auch Hürden und Belastungsfaktoren berichtet, etwa Dokumentationspflichten und Eingriffe in Praxisabläufe.

**Diskussion:**

Um die Attraktivität des Innovationsfonds für die hausärztliche Versorgung zu erhöhen, gilt es, die Hausarztkonformität von Projekten umfassend sicherzustellen, v. a. mit Blick auf ärztliche Entscheidungsspielräume, die Limitierung von Dokumentationspflichten, die Gewährleistung von Praxisroutinen, eine stärkere Involvierung in die Forschungsplanung sowie eine Aufwertung des hausärztlichen Settings.

**Zusatzmaterial online:**

Zusätzliche Informationen sind in der Online-Version dieses Artikels (10.1007/s00103-022-03533-y) enthalten.

## Abstract (en)

**Background:**

In 2015, the German Innovation Fund was established to promote improvements in the quality of medical care. In order for new care models and interventions to be tested and incorporated into standard care in the future, primary care must be included.

**Objectives:**

The study explores general practitioners’ (GPs’) attitudes, participation-relevant expectations, and experiences with regard to Innovation Fund projects.

**Methods:**

Between July and October 2021, all 13,170 general practitioners in Baden-Württemberg, Hesse, and Rhineland-Palatinate were invited to take part in an online survey. Included in the evaluation were 3556 completed questionnaires (response rate: 27%). In addition to the descriptive analysis, a *t*-test on independent samples was used to determine significant differences between two groups.

**Results:**

Of the respondents, 83% were familiar with the Innovation Fund. Most of the respondents associate it with opportunities and potential (including intensification of application-oriented healthcare research, independent financing, and inclusion of primary care). Nevertheless, many GPs are unsure to what extent primary care can benefit from the Innovation Fund in the longer term. When it comes to willingness to participate in Innovation Fund studies, the respondents are divided. Respondents who have already participated in such projects (24%) draw a positive balance (benefit of the intervention, cost-benefit ratio). However, hurdles and stress factors are also reported, such as documentation requirements and interventions in practice processes.

**Conclusions:**

In order to increase the attractiveness of the Innovation Fund for GPs, it is important to ensure that projects are fully compliant with primary care, especially with regard to the scope of medical decision-making, the limitation of documentation obligations, the guarantee of practice routines, a greater involvement in research planning, and an upgrading of the GP setting.

## Einleitung

Zur Optimierung der medizinischen Versorgung ist es längerfristig erforderlich, neue Versorgungsformen zu konzipieren bzw. zu erproben sowie die theoretische und anwendungsbezogene Versorgungsforschung zu stärken [1, 2]. Zudem bedarf komplexe klinische Forschung oftmals einer unabhängigen und stabilen Finanzierung. Um diesen Anforderungen Rechnung zu tragen, wurde im Jahr 2015 der beim Gemeinsamen Bundesausschuss (G-BA) angesiedelte Innovationsfonds verankert [3]. Im Sinne eines gesundheitspolitischen Instruments zielt er darauf ab, durch Entwicklung neuer Versorgungskonzepte eine evidenzbasierte Weiterentwicklung der umlagefinanzierten Gesundheitsversorgung voranzutreiben [2–5]. In der derzeitigen Förderphase stehen jährlich 200 Mio. € an Fördervolumen bereit, die von den gesetzlichen Krankenkassen und dem Gesundheitsfonds bereitgestellt werden.

Gefördert wurden bislang z. B. Versorgungsmodelle in ländlichen Gebieten, für spezielle Patient*innengruppen, zur Arzneimitteltherapie sowie Modelle mit Delegation und zur Stärkung der sektorenübergreifenden Versorgung [6]. Eine wichtige Anforderung des Innovationsfonds ist es, dass geförderte Projekte geeignet sein müssen, im Hinblick auf die entwickelte Versorgungsform dauerhaft implementiert zu werden [3, 4, 7]. Hierzu müssen sie wissenschaftlich evaluiert werden und so die Wirksamkeit ihrer Intervention unter Beweis stellen. Bei Projekterfolg besteht die Aussicht auf Übernahme in die Regelversorgung oder Aufnahme in Selektivverträge [1, 7].

Für innovationsfondsgeförderte Interventionen und Versorgungskonzepte bildet die Primärversorgung einen wichtigen Dreh- und Angelpunkt. Eingedenk der Tatsache, dass neue Versorgungsmodelle v. a. im Niedrigprävalenzbereich mit einer ausreichend großen Anzahl an Patient*innen stattfinden müssen, um Effekte hinreichend belegen zu können, ist es oftmals erforderlich, mit dem hausärztlichen Setting zu kooperieren [8–10]. Theoretisch birgt der Innovationsfonds die Chance, die hausärztliche Versorgung besonders von neuen Versorgungsformen profitieren zu lassen [11]. Diesem Potenzial steht die Herausforderung gegenüber, eine ausreichende Zahl von niedergelassenen Allgemeinärzt*innen für solche Forschungsvorhaben zu gewinnen, die häufig ein anspruchsvolles clusterrandomisiertes Design („cluster randomized trials“ [CRT]) erfordern. Damit verbunden ist die Frage, inwieweit Innovationsfondsmodelle dem allgemeinmedizinischen Setting entgegenkommen und sich dort implementieren lassen. Um Einstellungen, Erfahrungswerte und mögliche Problemlagen in Bezug auf die Einbeziehung der Hausarztmedizin in Innovationsfondsprojekte zu adressieren, kommt es entscheidend darauf an, die Sicht von niedergelassenen Allgemeinärzt*innen zu beleuchten.

Bislang fehlt es an Studien, die untersuchen, welche Positionen Hausärzt*innen konkret in Bezug auf den Innovationsfonds vertreten. Neben der Frage, inwieweit sie den Innovationsfonds kennen und sich bereits an assoziierten Projekten beteiligt haben, ist insbesondere von Bedeutung, welche Erfahrungen sie mit einer Projektteilnahme gemacht haben und wie sie diese bilanzieren. Auch ist die Frage von Interesse, welche Sichtweisen Hausärzt*innen ggf. davon abhalten, sich an Innovationsfondsstudien zu beteiligen.

## Methodik

Um das skizzierte Forschungsdesiderat zu adressieren und ein aussagekräftiges Meinungs- und Erfahrungsbild hausärztlich tätiger Mediziner*innen zu gewinnen, wurde bei der vorliegenden Studie ein explorativer Ansatz verfolgt. Aufbauend auf 2 als Vorstudie fungierenden Fokusgruppendiskussionen mit jeweils 10 Hausärzt*innen^1^ im Frühjahr 2021, erfolgte eine breit angelegte Befragung von Hausärzt*innen in 3 Bundesländern. Die Wahl fiel auf eine Onlinebefragung mit schriftlich-postalischem Anschreiben.

### Erhebungsinstrument

Der Fragebogen (siehe Onlinematerial) wurde unter Berücksichtigung der Diskussionsergebnisse, eigener Forschungs- und Rekrutierungserfahrungen der Autoren im Innovationsfondskontext [12, 13] sowie einer Literaturrecherche (u. a. Lech et al. [11] und Heytens et al. [4]) entwickelt. Er setzt sich aus 5 inhaltlichen Blöcken zusammen:

- Bekanntheit des Innovationsfonds,
- Teilnahmebereitschaft und deren Voraussetzungen,
- Einstellungen in Bezug auf Innovationsfondsprojekte und deren Nutzen,
- Erfahrungen bei Teilnahme an spezifischen Projekten,
- wahrgenommene Optimierungspotenziale.

Die Ergebnisse der Fokusgruppendiskussionen flossen in Verbindung mit der Literaturanalyse v. a. bei der Erstellung der verwendeten Itembatterien ein (Frage 4, 9).

Neben den standardisierten Fragen wurden mehrere offene Fragen eingesetzt (Frage 3, 6, 16, 17, 22, 23). Als soziodemografische Merkmale wurden Geschlecht, Alter, Praxisumgebung, Praxisform und Anzahl der Patient*innen pro Quartal erhoben. Vor dem Feldeinsatz wurde ein Pretest durchgeführt.

### Rekrutierung und Stichprobe

Auf schriftlich-postalischem Weg zur Teilnahme an der anonymisierten Befragung eingeladen wurden zwischen Juli und Oktober 2021 sämtliche 13.170 als Behandler*innen aktive Hausärzt*innen in Baden-Württemberg (6664), Hessen (3839) und Rheinland-Pfalz (2667). Es handelte sich um ein einmaliges Anschreiben, in dem die zu befragenden Ärzt*innen u. a. einen passwortgeschützten Zugang zur Onlinebefragung mitgeteilt bekamen. Anreize (Incentives) zur Teilnahme gab es nicht.

Von den 3595 bearbeiteten Fragebögen gingen 3556 vollständig ausgefüllte Bögen in die Auswertung ein (Rücklauf: 27 %). Tab. 1 stellt die gewonnene Stichprobe den repräsentativen Daten der Kassenärztlichen Vereinigung (KV) zum Aufbau der Hausärzteschaft in Deutschland gegenüber.

**Table 1:** 

	Stichprobe (*N* = 3556)	Repräsentativstatistik
Geschlecht:	62 % männlich	58 % männlich
	38 % weiblich	42 % weiblich^a^
Durchschnittsalter:	55 (Median: 55)	56 (Median: 57)^a^
Praxisumgebung:	51 % mittel- und großstädtisch	41 % mittel- und großstädtisch
	49 % ländlich-kleinstädtisch	59 % ländlich-kleinstädtisch^a^
Praxisform:	61 % Einzelpraxen	56 % Einzelpraxen
	32 % Gemeinschaftspraxen	38 % Gemeinschaftspraxen
	7 % MVZ/sonstige Einrichtung	6 % MVZ/sonstige Einrichtung^b^
Patient*innen pro Quartal:	25 % 500–1500, 28 % 1501–2000, 47 % > 2000	Keine vollständigen Daten verfügbar
Mitgliedschaft in einem Ärzt*innennetzwerk:	445	Keine vollständigen Daten verfügbar

*MVZ* medizinisches Versorgungszentrum

^a^Basierend auf den Versorgungsforschungsdaten der Kassenärztlichen Vereinigung (KV) für Rheinland-Pfalz (Stand: 31.12.2020), abrufbar unter: https://www.kv-rlp.de/institution/engagement/versorgungsforschung/

^b^Basierend auf den KV-Versorgungsforschungsdaten für Deutschland (Stand: 31.12.2020), abrufbar unter: https://gesundheitsdaten.kbv.de/

### Datenanalyse

Die Daten wurden mittels SPSS 23.0 (VERBI Software Consult GmbH, Berlin) ausgewertet. Zur Feststellung von signifikanten Unterschieden zwischen 2 Gruppen kam ein *t*-Test bei unabhängigen Stichproben zum Einsatz. Die Auswertung der offenen Fragen basiert auf einer Nachcodierung im Sinne der qualitativen Inhaltsanalyse und beinhaltete die Erstellung eines Kategoriensystems [14]. Als Reporting Statement wurde STROBE herangezogen.

## Ergebnisse

### Bekanntheit des Innovationsfonds

83 % der Befragten ist der Innovationsfonds bekannt. Dass auch vertragsärztlich tätige Hausärzt*innen an entsprechenden Projekten partizipieren und Patient*innen einschreiben können, ist 64 % bekannt. Während 94 % der in Groß- und Mittelstädten ansässigen Ärzt*innen über den Innovationsfonds informiert sind, beträgt dieser Anteil unter Ärzt*innen in Kleinstädten und Landgemeinden 70 % (*p* < 0,001). Über die Möglichkeit, dass auch hausärztliche Praxen an Innovationsprojekten teilnehmen können, sind 79 % der städtischen Ärzt*innen in Kenntnis, gegenüber 47 % der Landärzt*innen (*p* < 0,001).

### Teilnahmebereitschaft und Voraussetzungen

Für 31 % der Befragten käme es prinzipiell infrage, in Zukunft an einem Innovationsfondsprojekt teilzunehmen; weitere 24 % geben an, bereits an mindestens einer assoziierten Studie partizipiert zu haben. 45 % bekunden, eine Teilnahme an einem Innovationsfondsprojekt komme für sie nicht infrage. Unter Ärzt*innen unterhalb des Durchschnittsalters von 55 Jahren geben 47 % an, dass eine Innovationsfondsteilnahme infrage käme, im Vergleich zu 20 % der Ärzt*innen ab 55 Jahre (*p* < 0,001).

Als Begründung für ihre Teilnahmebereitschaft führen die entsprechenden Ärzt*innen in einer offenen Nachfrage v. a. Neugier und Mitwirkung in Bezug auf wissenschaftliche Forschung (35 %) sowie den Wunsch an, zu einer besseren Versorgung und Lebensqualität von Patient*innen beizutragen (45 %). Befragte, für die eine Teilnahme nicht infrage kommt, begründen dies mit hoher Arbeitsbelastung (54 %), Sorge vor Überbeanspruchung bei Teilnahme an Forschungsaktivitäten (44 %) sowie teilweise mit Skepsis in Bezug auf den Nutzen von wissenschaftlicher Forschung (29 %).

Für jene Ärzt*innen, für die eine Innovationsfondsteilnahme infrage käme oder die bereits an einem oder mehreren Projekten partizipiert haben, spielen als Voraussetzungen neben dem absehbaren Nutzen in der Patient*innenversorgung Fragen der (begrenzten) Mehrbelastung, der angemessenen Honorierung sowie der strukturellen Aufwertung der hausärztlichen Tätigkeit eine entscheidende Rolle (Tab. 2). Ebenfalls wichtig ist den Befragten, dass Projekte keine größeren Veränderungen von Arbeitsabläufen und Zuständigkeiten innerhalb der Praxis bewirken. Vergleichsweise weniger von Interesse sind für die Befragten Aspekte der Effizienzsteigerung in der Patient*innenversorgung (z. B. durch gezielten, evidenzbasierten Ressourceneinsatz) oder der größeren Kosteneffizienz im Gesundheitssystem. Ähnliches gilt für Schulungsmaßnahmen zur Erweiterung diagnostischer oder therapeutischer Kompetenzen (z. B. im Hinblick auf Evidenzbasierung oder Leitlinienorientierung).

**Table 2:** 

Fragewortlaut:* Welche der folgenden Aspekte wären für Sie als Voraussetzungen besonders wichtig, damit es für Sie in Frage kommt, an einem Innovationsfonds-Projekt teilzunehmen? Bitte geben Sie max. 5 Punkte an *(*N* = 1986)	Zustimmung (in %)
Der (Mehr‑)Aufwand bei einer Teilnahme muss überschaubar bleiben (z. B. Vor- und Nachbereitung, Dokumentationsaufwand)	68
Es muss eine angemessene Vergütung für teilnehmende Hausärzt*innen geben	59
Die hausärztliche Rolle und Stellung im Gesundheitswesen sollte durch das Projekt gestärkt und Hausärzt*innen in ihrer Bedeutung aufgewertet werden	57
Es muss ein klarer diagnostischer bzw. therapeutischer Nutzen und Versorgungsvorteil für meine Patient*innen erkennbar sein (z. B. erfolgreicheres Management multimorbider und chronisch kranker Patient*innengruppen, optimierte Diagnostik)	55
Das Projekt sollte erkennbar zu einer Vermeidung von Über- bzw. Unterversorgung beitragen	54
Die Teilnahme am Projekt darf keine nennenswerte Veränderung von Arbeitsabläufen, Routinen und Zuständigkeiten innerhalb der Praxis (Praxismanagement) bewirken	42
Das Projekt sollte helfen, Versorgungskrisen frühzeitig zu antizipieren und durch präventive Maßnahmen zu verhindern (z. B. Dekompensation von Demenzerkrankten und/oder deren Angehörigen)	28
Das Projekt sollte die interdisziplinäre, multiprofessionelle Versorgung stärken und diese mit der Hausarztmedizin (besser) verzahnen	26
Die Zusammenarbeit zwischen verschiedenen Versorgungsebenen bzw. Sektoren sollte durch das Projekt verbessert werden	25
Das Projekt sollte zu mehr Effizienz in der Patient*innenversorgung beitragen (z. B. durch gezielten Ressourceneinsatz, Beschränkung auf zentrale Indikatoren, Vermeidung von Mehrfachdokumentationen und doppelten Behandlungen)	18
Das Projekt sollte helfen, die individuellen Bedürfnisse von Patient*innen bzw. Patient*innengruppen besser zu berücksichtigen	17
Das Projekt sollte helfen, das diagnostische bzw. therapeutische Vorgehen bei der Versorgung bestimmter Patient*innengruppen besser zu strukturieren und zu regeln (z. B. durch klare Versorgungspfade)	8
Das Projekt sollte dazu beitragen, die Kosten für das Gesundheitssystem insgesamt zu senken	7
Im Rahmen des Projekts werden Hausärzt*innen geschult, sodass sie ihre diagnostischen bzw. therapeutischen Kompetenzen in einem Themengebiet erweitern können (z. B. im Hinblick auf Evidenzbasierung oder Leitlinienorientierung)	4

Während städtische Ärzt*innen stärker daran interessiert sind, dass Innovationsfondsprojekte die Zusammenarbeit zwischen den Versorgungsebenen optimieren (31 % gegenüber 14 % der Landärzt*innen, *p* < 0,001), wünscht sich ein größerer Anteil der Ärzt*innen im ländlichen Raum, dass solche Studien zu mehr Effizienz in der Patient*innenversorgung beitragen (32 % gegenüber 11 % der städtischen Ärzt*innen, *p* < 0,001). Ferner betonen Landärzt*innen erheblich häufiger, das Projekt dürfe die Praxisroutinen nicht verändern (64 % gegenüber 30 % der städtischen Ärzt*innen, *p* < 0,001).

### Einstellungen in Bezug auf Innovationsfondsprojekte und Nutzen für die Versorgung

49 % der Befragten beurteilen die Einrichtung des Innovationsfonds grundsätzlich positiv, 19 % als negativ (32 % schwer zu sagen). Der Anteil derjenigen, die zu einem positiven Urteil gelangen, ist unter städtischen Ärzt*innen besonders groß (60 % gegenüber 38 % der Landärzt*innen). 38 % aller Befragten nehmen im Innovationsfonds für die Patient*innenversorgung einen (sehr) großen Nutzen wahr, weitere 24 % einen geringen Nutzen (10 % kein Nutzen, 28 % schwer zu sagen).

Die Befragten, die eingangs der Befragung angaben, den Innovationsfonds zu kennen, erhielten eine Itembatterie mit Zitaten (Tab. 3). Diese wurden aus den vorangegangenen Fokusgruppendiskussionen extrahiert. Es zeigt sich, dass ein großer Teil der Befragten den Innovationsfonds mit Chancen für das Gesundheitswesen assoziiert, besonders mit Blick auf eine unabhängige Finanzierung und die Identifizierung bzw. Schließung von Versorgungslücken. Ebenfalls wird von einer Mehrheit begrüßt, dass Hausärzt*innen sich nun tendenziell stärker in wissenschaftliche Forschung einbringen können. Zugleich artikulieren Teile der Befragten die Sorge vor negativen Effekten, z. B. in Bezug auf die Adressierung hausärztlicher Versorgungsbedarfe. Ein Teil der Befragten betrachtet den Innovationsfonds als stark politisch gesteuerte Einrichtung und ist sich nicht sicher, inwieweit geförderte Versorgungsmodelle letztlich den Weg in die Regelversorgung finden werden. Vergleichsweise gering ist der Zuspruch der Befragten mit Blick auf die Aussicht, dass Innovationsfondsprojekte längerfristig zu einer Aufwertung der hausärztlichen Versorgung führen.

**Table 3:** 

Fragewortlaut: Als Hausärzt*in kann man ganz unterschiedliche Positionen in Bezug auf den Innovationsfonds vertreten. Hier stehen verschiedene Aussagen, die wir im Rahmen einer Interviewvorstudie mit Hausärzt*innen erfasst haben. Bitte geben Sie an, inwiefern Sie der jeweiligen Aussage zustimmen (*N* = 2983)	Stimme voll und ganz zu (%)	Stimme eher zu (%)	Stimme eher nicht zu (%)	Stimme gar nicht zu (%)	Schwer zu sagen, weiß nicht (%)
*Der Innovationsfonds ist positiv zu sehen, weil durch ihn komplexe klinische und Versorgungsforschung unabhängig finanziert werden kann*	38	22	15	13	12
Innovationsfonds-Projekte haben das Potenzial, nachhaltige Verbesserungen in der Patient*innenversorgung zu bewirken	23	34	12	21	10
Der Innovationsfonds bietet Hausärzt*innen die Chance, sich stärker in wissenschaftliche Forschung wie klinische Studien einzubringen	29	26	8	17	20
*Innovationsfonds-Projekte führen zu einer Verkomplizierung des Gesundheitswesens, z.* *B. weil neue Akteure oder Strukturen geschaffen werden*	15	34	16	16	19
*Der Innovationsfonds ist v.* *a. eine politisch gewollte Einrichtung; es werden nicht unbedingt Projekte gefördert, die das Gesundheitswesen braucht*	26	22	17	18	17
*Innovationsfonds-Projekte gehen häufig an der hausärztlichen Versorgungsrealität vorbei*	16	20	15	23	26
*Ich bezweifle, dass Innovationsfonds-Projekte am Ende den Weg in die Regelversorgung finden*	17	19	16	30	18
*Innovationsfonds-Projekte werden mit ihren Interventionen längerfristig zu einer Aufwertung der hausärztlichen Versorgung führen*	14	21	20	25	20

Ärzt*innen in städtischen Gebieten stimmen der letzteren Aussage erheblich häufiger zu als Landärzt*innen (51 % zu 28 %, *p* < 0,001). Unter Hausärzt*innen, für die nicht vorstellbar ist, an Innovationsfondsstudien teilzunehmen, bezweifelt eine Mehrheit, dass Innovationsfondsmodelle den Weg in die Regelversorgung finden werden (57 %), und erwartet nur in geringem Maße eine Aufwertung des hausärztlichen Settings (58 %).

### Erfahrungen bei Teilnahme an spezifischen Projekten

Innerhalb der Stichprobe wurden 45 % der Befragten noch nie in Bezug auf eine mögliche Innovationsfondsteilnahme angesprochen oder zu rekrutieren versucht. Unter den übrigen Befragten geben 19 % an, dies sei einmal bzw. bei 14 % zweimal vorgekommen; 15 % nennen 3–5 und 7 % mehr als 5 Rekrutierungsversuche.

24 % (875) der Befragten haben nach eigener Aussage bereits an einem oder mehreren Innovationsfondsprojekten teilgenommen oder tun dies aktuell. Die Gruppe der Teilnehmenden beinhaltet zu 92 % städtische Ärzt*innen und zu 8 % Landärzt*innen. Von den 875 Befragten sind 33 % Einzelpraxen und 67 % Gemeinschaftspraxen. Hierunter befinden sich zu 73 % Personen unterhalb des Durchschnittsalters. 35 % der Projektteilnehmenden sind Mitglied eines Ärzt*innennetzwerks.

Aus den Angaben der Befragten ist erkennbar, dass bei dem Großteil der Projekte, an denen partizipiert wurde, die Optimierung der spezifischen Patient*innenversorgung, der Ausbau von regionalen und multiprofessionellen Versorgungsstrukturen sowie die Förderung von Evidenzbasierung bzw. Leitlinienadhärenz Schwerpunkte waren (Tab. 4). Ebenfalls zeigt sich ein hoher Anteil von Projekten mit telemedizinischem Bezug sowie dem Ziel, eine Delegation von Versorgungsleistungen zu ermöglichen. Seltener vertreten sind etwa Projekte zur Versorgung vulnerabler Gruppen (z. B. pflegende Angehörige) oder von Menschen mit Behinderung sowie zur Förderung von Gesundheits- bzw. Kommunikationskompetenz.

**Table 4:** 

Fragewortlaut:* Welchen der folgenden Themengebiete lässt sich das/die Innovationsfonds-Projekt(e), an dem/denen Sie beteiligt sind bzw. waren, zuordnen? (Mehreres kann angegeben werden.)* (*N* = 875)	Zustimmung (in %)
Versorgung für spezifische Krankheiten/Krankheitsgruppen	65
Arzneimitteltherapie bzw. Arzneimitteltherapiesicherheit	58
Sektorenübergreifende bzw. -unabhängige Versorgung	58
Medizinische Leitlinien und deren (Weiter‑)Entwicklung	54
Geriatrische Versorgung	50
Telemedizin, Telematik, E‑Health/M-Health	48
Delegation und Substitution von Leistungen	47
Versorgung für spezielle Patient*innengruppen	39
Stärkung der regionalen Gesundheitsversorgung	32
Versorgung von vulnerablen Gruppen (z. B. pflegende Angehörige)	27
Kommunikation mit Patient*innen und Förderung der Gesundheitskompetenz	15
Versorgung in strukturschwachen bzw. ländlichen Gebieten	11
Versorgung für Menschen mit Behinderung	6
Sonstiges	6

42 % der teilnehmenden Hausärzt*innen geben an, selbst auf das Projekt aufmerksam geworden zu sein und sich z. B. als Reaktion auf das Lesen einer Bekanntmachung gemeldet zu haben. 36 % nennen eine Rekrutierung durch Projektmitarbeiter*innen oder -partner, wie z. B. Ärzt*innennetzwerke oder Krankenkassen (22 % teils, teils).

75 % der projektinvolvierten Befragten geben an, dass es infolge der Teilnahme erforderlich gewesen sei, Mitglieder des Praxisteams zu schulen. Dies war v. a. bei Ärzt*innen der Fall, die an Projekten zur Arzneimitteltherapie und Versorgung spezifischer Krankheiten teilgenommen haben. 80 % der Befragten bekunden, dass es aufgrund der Teilnahme an einem oder mehreren Innovationsfondsprojekten im Praxisbetrieb zu starken (27 %) oder moderaten (53 %) Komplikationen gekommen sei (20 % keine Komplikationen).

### Bilanzierung und Optimierungspotenziale

Insgesamt ziehen die Befragten, die an Innovationsfondsprojekten mitgewirkt haben, ein positives Fazit in Bezug auf den Nutzen der erprobten Intervention. So urteilen 72 %, die Versorgung bzw. Therapie der einbezogenen Patient*innen habe sehr stark (13 %) oder eher stark (45 %) profitiert (18 % eher nicht so stark, 16 % überhaupt nicht, 8 % schwer zu sagen). Analog bilanzieren 66 %, der Nutzen der Projektteilnahme habe deutlich (43 %) oder etwas (23 %) gegenüber dem Aufwand überwogen (11 % in etwa gleich, 12 % Aufwand überwog etwas, 11 % Aufwand überwog deutlich). Projekte in den Themenfeldern Versorgung in strukturschwachen Gebieten, Arzneimitteltherapie/-sicherheit, Delegation und Substitution sowie sektorenübergreifende Versorgung werden hinsichtlich ihres Mehrwertes am positivsten beurteilt.

Auf eine offene Frage antworten die projektinvolvierten Ärzt*innen, sie seien besonders mit den beobachteten (Therapie‑)Resultaten bzw. der optimierten Patient*innenversorgung (69 %), der besseren Kooperation mit anderen Versorgungsakteuren und Sektoren (52 %) und der Erweiterung diagnostischer bzw. therapeutischer Kompetenzen (40 %) zufrieden. Als negativ wurden Dokumentationspflichten (z. B. Einschreibung von Patient*innen) und ein oftmals hoher bürokratischer Aufwand (64 %) erlebt, die Beeinträchtigung von Praxisroutinen aufgrund der Projektteilnahme (55 %) sowie eine aus Sicht mancher Ärzt*innen zu geringe Einbeziehung in Forschungsprozesse und projektbezogene Entscheidungen (43 %).

15 % aller Befragten, die bereits an Innovationsfondsprojekten partizipiert haben, geben an, ihre Teilnahme schon einmal abgebrochen zu haben. Als Begründung hierfür werden v. a. eine zu hohe Mehrbelastung, der (dokumentarische) Aufwand und eine Kompromittierung von Praxisabläufen angegeben. Ebenfalls genannt werden zu geringe Entscheidungs- und Mitwirkungsmöglichkeiten.

In der Gruppe derjenigen Ärzt*innen, die an Innovationsfondsprojekten teilgenommen haben, wünschen sich die Befragten eine Reihe von Verbesserungen. Diese beziehen sich auf eine Begrenzung von Dokumentationspflichten (65 %), mehr organisatorische Klarheit in Bezug auf die Projektkoordination (56 %), die Ermöglichung von mehr ärztlicher Entscheidungsflexibilität (z. B. bei der Patient*inneneinbestellung und therapiebezogenen Entscheidungen), weniger starke Eingriffe in Praxisabläufe (49 %) sowie die Stärkung und bessere Strukturierung der Kommunikation bzw. Kooperation an den Schnittstellen mit anderen Versorgungsakteuren (37 %). Nicht zuletzt sprechen sich die Ärzt*innen für eine aufwandsgerechte(re) Honorierung aus (34 %).

Aus den Antworten geht hervor, dass die Position von Hausärzt*innen in verschiedenen Etappen von Innovationsfondsprojekten weiter gestärkt werden sollte. 57 % aller Befragten ist es wichtig, dass Hausärzt*innen stärker als bislang in die Konzeption und Entwicklung neuer Studien im Innovationsfondskontext einbezogen werden.

## Diskussion

Die Ergebnisse der Befragung zeigen, dass der Innovationsfonds inzwischen unter einer Mehrheit der Hausärzt*innen breite Bekanntheit erlangt hat. Hinsichtlich der Bereitschaft zur Mitwirkung an Modellvorhaben zeigen sich die Befragten gespalten. Jene Ärzt*innen, die sich eine Mitwirkung grundsätzlich vorstellen können oder bereits an Forschungsprojekten partizipieren, stellen diesbezüglich eine Reihe von Anforderungen. Jenseits des Mehrwerts für die Patient*innenversorgung werden hierbei u. a. eine handhabbare Mehrbelastung, eine nicht zu starke Tangierung von Praxisroutinen sowie eine strukturelle Aufwertung der hausärztlichen Tätigkeit hervorgehoben. Dies deckt sich mit Resultaten vorangegangener Befragungen von (aufzubauenden) hausärztlichen Forschungspraxennetzen (z. B. [10, 15–20, 36]). In einer Studie zeigten Güthlin et al., dass allgemeinärztliche Praxen v. a. dann Interesse an komplexen Forschungsprojekten haben, wenn das Thema für sie relevant erscheint und ein konkreter Nutzen für Team und Patientenschaft erkennbar ist. In diesem Zusammenhang ist wenig überraschend, dass Ärzt*innen, die an Innovationsfondsmodellen partizipiert haben, Studien in Themenfeldern wie ländliche Versorgung, Delegation oder Sektorenkooperation besonders günstig bilanzieren. Darüber hinaus wünschen viele Hausärzt*innen heute nicht, „dass man als Praxis nur ‚beforscht‘ wird“, sondern möchten eine Mitgestaltung bei der Umsetzung von Forschungsvorhaben übernehmen [15, S. 180, 16].

Insgesamt fällt auf, dass die Positionen von Hausärzt*innen in Bezug auf den Innovationsfonds positiv ausfallen und Chancen des Konzepts wahrgenommen werden, z. B. mit Blick auf eine Intensivierung anwendungsnaher Versorgungsforschung oder die Einbeziehung der hausärztlichen Versorgung in klinische Forschung. Auf der anderen Seite ist eine z. T. kritisch-distanzierte Haltung festzustellen, wenn es um die langfristige Zielorientierung entsprechender Studien geht. Ein Teil der Befragten ist unsicher, inwiefern durch Innovationsfondsmodelle geschaffene Strukturen tatsächlich zu einer dauerhaften Effektivierung des Gesundheitswesens beitragen können. Auch besteht Unsicherheit, ob die hausärztliche Versorgung auf lange Sicht tatsächlich von derartigen Forschungsbeteiligungen profitieren kann.

Während städtische Ärzt*innen im Sample deutliche Vorzüge im Innovationsfonds ausmachen, sind Landärzt*innen zurückhaltender. Dies deckt sich mit der allgemeinen Forschungslage, wonach hausärztliche Mediziner*innen in ländlichen Regionen geringere Mehrwerte evidenzbasierter Strukturen bzw. Instrumente wahrnehmen [21, 22]. Analog dazu befinden sich unter den 24 % der Befragten, die bereits an Innovationsfondsprojekten teilgenommen haben, überwiegend im städtischen Umfeld angesiedelte Ärzt*innen, wo Versorgungsstrukturen ausdifferenzierter sind, die häufig eine Voraussetzung für die Realisierung von klinischer Forschung sind [8, 10].

Die Bilanz, die infolge der Teilnahme an den jeweiligen Studien gezogen wird, ist mehrheitlich positiv. Dies gilt sowohl mit Blick auf die Versorgung bzw. Steigerung der Therapiequalität der einbezogenen Patient*innen als auch bezüglich des Verhältnisses von Aufwand und Nutzen. Zugleich schildern Teile der Befragten negative Erfahrungen und Belastungsfaktoren, die sich in Dokumentationspflichten und Verwaltungsaufwand, einer Veränderung von Praxisabläufen und einer nicht immer aufwandsadäquaten Honorierung niederschlagen.

Die Resultate der Befragung können als Bestätigung gewertet werden, dass Hausärzt*innen heute in stärkerem Maße bereit sind, sich an empirischen Studien zur Optimierung von Versorgung zu beteiligen. Gerade jüngere Allgemeinmediziner*innen im urbanen Einzugsgebiet fundieren ihre Arbeit heute vermehrt auf standardisierten, evidenzorientierten Interventionen [21, 22]. Dennoch steht ein erheblicher Teil der allgemeinmedizinischen Praxen solchen Forschungsvorhaben nicht zur Verfügung oder bleibt zurückhaltend [8–11]. Damit kommt es regional zu einer Knappheit an für komplexe Studien rekrutierbaren Hausärzt*innen, wie gerade Projekterfahrungen aus dem Innovationsfondskontext belegen; oft wurden ursprünglich angestrebte Ärzt*innen- und Patient*innenzahlen nicht erreicht [23]. Ein Beispiel hierfür zeigt die Arbeit von Lech et al. [11], die über eine clusterrandomisierte Studie zur Optimierung der ambulanten Demenzversorgung berichten. Trotz einer großen Bandbreite von Rekrutierungsstrategien berichten die Autor*innen über Schwierigkeiten, niedergelassene Allgemeinärzt*innen zu gewinnen.

Obwohl die Gründe für die Hürden und Herausforderungen, Hausärzt*innen für Innovationsfondsstudien zu rekrutieren, bis dato kaum untersucht wurden, bestehen Hinweise, dass sich entsprechende Projekte nicht immer reibungslos mit dem hausärztlichen Versorgungsalltag vereinbaren lassen. Neben dem Problem einer grundsätzlichen Zeit- bzw. Ressourcenknappheit [16] werden im allgemeinmedizinischen Fachumfeld Sorgen hinsichtlich logistischer Machbarkeit und ausufernder Mehrbelastung artikuliert, aber auch gelegentlich Zweifel an der Integrierbarkeit bzw. Passgenauigkeit von Interventionen im hausärztlichen Praxisalltag und der Anreizstruktur von Innovationsfondsstudien [15, 16, 20, 23, 24].

Zwar sind Gesundheits- und Innovationssysteme nicht unmittelbar vergleichbar, jedoch zeigen Studien in anderen westlichen Ländern, dass auch hier die Gewinnung und Einbeziehung von Hausärzt*innen in klinische Forschungsprojekte mit Problemen verknüpft ist [25–34]. In einer Übersichtsarbeit identifizieren Fletcher et al. [35] Barrieren wie schlechte Kommunikation durch Studienkoordinator*innen, hausärztliche Schwierigkeiten beim Verständnis von Forschungsmethoden, Bedenken hinsichtlich möglicher Schäden für Patient*innen und das Gefühl, von zu vielen Forschungsanfragen überfordert zu sein, ohne als echte Forschungspartner*innen wahrgenommen zu werden. Für Deutschland kommt im Unterschied zu anderen Ländern hinzu, dass es keine lange Tradition gibt, Hausärzt*innen in klinische Forschungsaktivitäten einzubeziehen [29, 36].

Über die angeführten Problematiken hinaus diskutieren Lech et al. [11] Anforderungen an eine spezifische Rekrutierung von hausärztlich tätigen Mediziner*innen. So wird hervorgehoben, dass diese bei CRT-Studien effektiviert werden könnte, wenn es eine stärkere Konzentration auf (regionale) Ärzt*innennetze gäbe, da hier wissenschaftliches Interesse, ein spezifischer Themenbezug und eine enge Abstimmung der teilnehmenden Ärzt*innen eher gegeben seien [18]. Auch in der vorliegenden Befragung war ein beträchtlicher Teil der in Studien involvierten Mediziner*innen Mitglied in einem Ärzt*innennetz.

Die von den Befragten artikulierten Verbesserungswünsche und Voraussetzungen für eine Beteiligung an Innovationsfondsstudien gehen konform mit den Ergebnissen vorangegangener Studien und zeigen Perspektiven auf, wie in Zukunft mehr Hausärzt*innen gewonnen werden könnten. So wünschen sich Allgemeinmediziner*innen nicht nur mit Blick auf die Teilnahme an Forschungsaktivitäten, sondern auch hinsichtlich der Partizipation an evidenzbasierten Strukturen bzw. Instrumenten (u. a. Disease-Management-Programme, Leitlinien) individuelle Entscheidungsflexibilität, die Limitierung von Verwaltungsaufwand und die Aufrechterhaltung eingefahrener Praxisroutinen [12, 20–22]. Zudem besteht bei einer Reihe von Hausärzt*innen der Wunsch, Projektaktivitäten (stärker) mitgestalten zu können. All dies verweist auf eine übergeordnete Hausarztkonformität von klinischen Forschungsprojekten, die bis dato nicht immer gegeben ist [15, 24, 29].

### Stärken und Schwächen

Die vorliegende Studie ist die erste Arbeit, die sich mit der Frage nach Einstellungen, Akzeptanz, Erwartungen und Erfahrungen von Hausärzt*innen mit Blick auf Innovationsfondsstudien befasst. Aufgrund der begrenzten Fallzahl und des regionalen Rekrutierungsschwerpunktes kann die Studie keinen repräsentativen Anspruch erheben. Zudem ist festzustellen, dass der Anteil an Hausärzt*innen mit Innovationsfondserfahrung in der Stichprobe verglichen mit der Gesamtheit der Hausärzteschaft erkennbar überrepräsentiert ist (Selection Bias). Insofern hat die Befragung thematisch interessierte bzw. engagierte Ärzt*innen verstärkt angesprochen, wohingegen davon auszugehen ist, dass Ärzt*innen ohne Bezug zu klinischer Forschung in geringerem Maße partizipiert haben. Entsprechend zu reflektieren sind auch die Angaben der Befragten zu den Themenschwerpunkten der jeweiligen Innovationsfondsprojekte (Tab. 4). So widerspiegelt die aufgeführte Rangfolge der Nennungen zwar das thematische Interesse der Hausärzt*innen, jedoch könnten diese Angaben auch durch die Zahl der für die einzelnen Themenfelder angebotenen Projekte beeinflusst sein.

Dennoch konnte eine heterogene Stichprobe gewonnen werden, die sich in wichtigen Merkmalen der Grundgesamtheit der Hausärzteschaft annähert (Tab. 1). Durch das explorative Vorgehen mit den vorgeschalteten Fokusgruppen und die Berücksichtigung offener Fragen konnte eine große Bandbreite hausärztlicher Perspektiven, Standpunkte und Erfahrungen erfasst werden.

Die vorliegende Studie hat sich nicht mit der Frage beschäftigt, inwieweit die Projekte, an denen die befragten Hausärzt*innen teilgenommen haben, von Instituten für Allgemeinmedizin (mit‑)durchgeführt oder koordiniert wurden. Diese Institute haben inzwischen viel Erfahrung in der Forschungszusammenarbeit mit Hausärzt*innen. Daher sollten künftige Studien ein Augenmerk darauf legen, ob bei einer Zusammenarbeit mit allgemeinmedizinischen Instituten die Studienbedingungen für die Hausärzteschaft ggf. günstiger ausfallen.

Ebenfalls wurde im Zuge der vorliegenden Befragung nicht erhoben, inwieweit es abseits von klinischen Studien im Innovationsfondskontext andere Settings gäbe, die der hausärztlichen Versorgung mit Blick auf die Bereitschaft zum wissenschaftlichen Engagement besser entgegenkämen. Arbeiten aus der allgemeinmedizinischen Versorgungsforschung legen nahe, dass das Modell der Forschungspraxis möglicherweise bessere Rekrutierungs- und Mitwirkungserfolge erzielen könnte [15, 18]. Insofern könnten die Ergebnisse der vorliegenden Arbeit perspektivisch den Resultaten einer hier anzuregenden Befragung gegenübergestellt werden, die Erfahrungen von Hausärzt*innen speziell im Forschungspraxenkontext breitflächig erfasst. Angesichts kürzlich entstandener größerer Forschungsnetze, die von allgemeinmedizinischen Instituten koordiniert werden, wäre eine solche Befragung in größerem Stil realisierbar.

### Schlussfolgerungen

Anhand der Ergebnisse der Befragung von 3556 Hausärzt*innen lassen sich Rückschlüsse ziehen, wie Hausärzt*innen insgesamt sowie aus bestehender Projekterfahrung den Innovationsfonds wahrnehmen und welche Anforderungen Studien erfüllen müssen, damit eine Teilnahme für Hausärzt*innen infrage kommt. So gilt es insbesondere, die Hausarztkonformität von Projekten umfassend sicherzustellen, v. a. mit Blick auf ärztliche Entscheidungsspielräume, die Limitierung von Dokumentationspflichten, die Beeinträchtigung von Praxisroutinen und eine stärkere Involvierung in die Forschungsplanung und eine längerfristige Aufwertung des hausärztlichen Settings. Innovationsfondsprojekte sollten so ausgestaltet und kommuniziert werden, dass eine klare Relevanz für den hausärztlichen Alltag erkennbar ist.

## Fazit

Für neue Versorgungsmodelle im Rahmen des Innovationsfonds bildet die hausärztliche Versorgung einen wichtigen Bezugspunkt, damit eine breite Patientenschaft erreicht und Effekte von Interventionen belegt werden können. Hausärzt*innen verbinden den Innovationsfonds mit Chancen und Potenzialen einer Optimierung der allgemeinmedizinischen Versorgung. Dennoch sind viele unsicher, inwiefern die Primärversorgung längerfristig vom Innovationsfonds wird profitieren können. Die subjektive Bilanz, die infolge der Teilnahme an den Versorgungsmodellen gezogen wird, ist mehrheitlich positiv (Steigerung der Therapiequalität, Aufwand-Nutzen-Verhältnis).

Um die Teilnahmebereitschaft von Hausärzt*innen an Innovationsfondsprojekten zu erhöhen, gilt es, die Hausarztkonformität von Projekten sicherzustellen, v. a. mit Blick auf ärztliche Entscheidungsspielräume, die Limitierung von Dokumentationspflichten, die Gewährleistung von Praxisroutinen, eine stärkere Involvierung in die Forschungsplanung sowie eine Aufwertung des hausärztlichen Settings.

## Supplementary Information





## Footnotes

1 Bei der Zusammenstellung der Fokusgruppen wurde darauf geachtet, dass allen Hausärzt*innen der Innovationsfonds bekannt war. Zudem wurden in jede Fokusgruppe mehrere Hausärzt*innen aufgenommen, die bereits Projekterfahrungen gemacht hatten.
