# Supplementary material for: The German Innovation Fund and primary care—What expectations and experiences do general practitioners have with regard to participating in innovative care models?
Source: Bundesgesundheitsblatt Gesundheitsforschung Gesundheitsschutz. 2022 Apr 27;65(6):697–705. [Article in German] doi: 10.1007/s00103-022-03533-y (PMC9132806; doi:10.1007/s00103-022-03533-y)
Supplement: Supplementary file 1 [file 103_2022_3533_MOESM1_ESM.pdf]

- Fragebogen: Hausärztliche Einstellungen zu und Erfahrungen mit Innovationsfonds-Studien -

1. Im Jahr 2016 wurde der sogenannte Innovationsfonds des Gemeinsamen Bundesausschusses (G-BA) eingerichtet, der zur Verbesserung der medizinischen Versorgung innerhalb der gesetzlichen Krankenversicherung neue Versorgungsformen und die Versorgungsforschung finanziell fördert. In der derzeitigen Förderphase stehen jährlich 200 Millionen Euro an Fördervolumen bereit, die von den gesetzlichen Krankenkassen und dem Gesundheitsfonds getragen werden. Gefördert wurden u.a. Versorgungsmodelle in strukturschwachen oder ländlichen Gebieten, für spezielle Patient*innen-gruppen (z.B. ältere Menschen, Menschen mit psychischen und/oder seltenen Erkrankungen), zur Arzneimitteltherapie oder Modelle mit Delegation. Insbesondere werden Projekte gefördert, die zur Verbesserung der sektorenübergreifenden Versorgung beitragen und ein Umsetzungspotential besitzen sowie Projekte, deren Ziel in der dauerhaften Weiterentwicklung der selektivvertraglichen Versorgung besteht. Bei Projekterfolg und nachgewiesener Wirksamkeit der entsprechenden Interventionen besteht die Aussicht auf Übernahme in die Regelversorgung oder Aufnahme in große Selektivverträge.

War Ihnen bekannt, dass es den Innovationsfonds gibt, oder ist Ihnen dies neu?

◯ War mir bekannt ◯ Ist mir neu

2. Auch vertragsärztlich tätige Hausärzt*innen können an Innovationsfondsprojekten teilnehmen und hierzu Patient*innen einschreiben. Die Teilnahme an Innovationsfondsprojekten ermöglicht Ihnen etwa, Ihren Patient*innen neue Versorgungsformen anzubieten (z.B. Unterstützung der Versorgung durch bestimmte diagnostische bzw. therapeutische Maßnahmen oder telemedizinische Geräte), die bislang nicht Teil der Regelversorgung sind. Je nach Projekt besteht dabei die Möglichkeit, die Versorgungsform Patient*innen nicht nur einer, sondern aller gesetzlichen Krankenkassen anzubieten. Darüber hinaus erhalten Hausärzt*innen für diese Form der Versorgung eine zusätzliche Vergütung.

War Ihnen bekannt, dass es Hausärzt*innen möglich ist, an Innovationsfonds-Projekten teilzunehmen, oder ist Ihnen dies neu?

◯ War mir bekannt ◯ Ist mir neu

3. Käme es grundsätzlich für Sie in Frage, an einem Innovationsfonds-Projekt teilzunehmen, oder war das vielleicht bereits der Fall, oder käme eine solche Teilnahme grundsätzlich nicht für Sie in Frage?

◯ Käme in Frage,
und zwar aus folgendem Grund/Gründen (einige Stichpunkte genügen):

____

____

◯ Habe bereits teilgenommen

◯ Käme grundsätzlich nicht in Frage, => bitte direkt zu **Frage 5** übergehen
und zwar aus folgendem Grund/Gründen (einige Stichpunkte genügen):

____

____

4. Welche der folgenden Aspekte wären für Sie als Voraussetzungen besonders wichtig, damit es für Sie in Frage kommt, an einem Innovationsfonds-Projekt teilzunehmen?
Bitte geben Sie max. 5 Punkte an.

◯	Es muss ein klarer diagnostischer bzw. therapeutischer Nutzen und Versorgungsvorteil für meine Patient*innen erkennbar sein (z.B. erfolgreicheres Management multimorbider und chronisch kranker Patientengruppen, optimierte Diagnostik).
◯	Der (Mehr-)Aufwand bei einer Teilnahme muss überschaubar bleiben (z.B. Vor- und Nachbereitung, Dokumentationsaufwand).
◯	Es muss eine angemessene Vergütung für teilnehmende Hausärzt*innen geben.
◯	Im Rahmen des Projekts werden Hausärzt*innen geschult, sodass sie ihre diagnostischen bzw. therapeutischen Kompetenzen in einem Themengebiet erweitern können (z.B. im Hinblick auf Evidenzbasierung oder Leitlinienorientierung).
◯	Die hausärztliche Rolle und Stellung im Gesundheitswesen sollte durch das Projekt gestärkt und Hausärzt*innen in ihrer Bedeutung aufgewertet werden.
◯	Die Zusammenarbeit zwischen verschiedenen Versorgungsebenen bzw. Sektoren sollte durch das Projekt verbessert werden.
◯	Das Projekt sollte die interdisziplinäre, multiprofessionelle Versorgung stärken und diese mit der Hausarztmedizin (besser) verzahnen.
◯	Das Projekt sollte zu mehr Effizienz in der Patient*innenversorgung beitragen (z.B. durch gezielten Ressourceneinsatz, Beschränkung auf zentrale Indikatoren, Vermeidung von Mehrfachdokumentationen und doppelte Behandlungen).
◯	Das Projekt sollte erkennbar zu einer Vermeidung von Über- bzw. Unterversorgung beitragen.
◯	Das Projekt sollte helfen, die individuellen Bedürfnisse von Patient*innen bzw. Patient*innengruppen besser zu berücksichtigen.
◯	Das Projekt sollte helfen, das diagnostische bzw. therapeutische Vorgehen bei der Versorgung bestimmter Patient*innengruppen besser zu strukturieren und regeln (z.B. durch klare Versorgungspfade)
◯	Die Teilnahme am Projekt darf keine nennenswerte Veränderung von Arbeitsabläufen, Routinen und Zuständigkeiten innerhalb der Praxis (Praxismanagement) bewirken.
◯	Das Projekt sollte dazu beitragen, die Kosten für das Gesundheitssystem insgesamt zu senken.
◯	Das Projekt sollte helfen, Versorgungskrisen frühzeitig zu antizipieren und durch präventive Maßnahmen zu verhindern (z.B. Dekompensation von Demenzerkrankten und/oder deren Angehörigen).
◯	Sonstiges, und zwar:

Bitte beantworten Sie die nachfolgenden Fragen nur, falls Ihnen der Innovationsfonds vor der Bearbeitung dieses Fragebogens bereits bekannt war *oder* Sie bereits an einem entsprechenden Innovationsfonds-Projekt teilgenommen haben bzw. dies aktuell tun. Ansonsten gehen Sie bitte direkt über zu Seite 7.

5. Ganz allgemein gefragt: Wie beurteilen Sie Innovationsfonds ganz grundsätzlich, wenn Sie zugrunde legen, was Sie bereits darüber gehört haben und ggf. eigene Erfahrungen mit bestimmten Projekten einbeziehen?

◯ Sehr gut ◯ Eher gut ◯ Eher schlecht ◯ Sehr schlecht ◯ Schwer zu sagen, weiß nicht

6. Warum sehen Sie das so? Einige Stichpunkte genügen.

____

____

____

7. Wie groß ist Ihrer Einschätzung nach der Nutzen, den der Innovationsfonds insgesamt für die Patient*innenversorgung in Deutschland hat?

◯ Sehr groß ◯ Eher groß ◯ Eher gering ◯ Kein Nutzen ◯ Schwer zu sagen, weiß nicht

8. Würden Sie sagen, dass sich Ihre grundlegende Einstellung zum Innovationsfonds in den letzten Jahren eher verbessert oder eher verschlechtert hat?

◯ Stark verbessert ◯ Etwas verbessert ◯ Gleich geblieben ◯ Eher verschlechtert ◯ Stark verschlechtert

9. Als Hausärzt*in kann man ganz unterschiedliche Positionen in Bezug auf den Innovationsfonds vertreten. Hier stehen verschiedene Aussagen, die wir im Rahmen einer Interviewvorstudie mit Hausärzt*innen erfasst haben. Bitte geben Sie an, inwiefern Sie der jeweiligen Aussage zustimmen.

	Stimme voll und ganz zu	Stimme eher zu	Stimme eher nicht zu	Stimme gar nicht zu	Schwer zu sagen, weiß nicht
„Innovationsfonds-Projekte haben das Potenzial, nachhaltige Verbesserungen in der Patient*innenversorgung zu bewirken."	◯	◯	◯	◯	◯
„Innovationsfonds-Projekte gehen häufig an der hausärztlichen Versorgungsrealität vorbei."	◯	◯	◯	◯	◯
„Der Innovationsfonds ist positiv zu sehen, weil durch ihn komplexe klinische und Versorgungsforschung unabhängig finanziert werden kann."	◯	◯	◯	◯	◯
„Der Innovationsfonds ist v.a. eine politisch gewollte Einrichtung; es werden nicht unbedingt Projekte gefördert, die das Gesundheitswesen braucht."	◯	◯	◯	◯	◯
„Innovationsfonds-Projekte werden mit ihren Interventionen längerfristig zu einer Aufwertung der hausärztlichen Versorgung führen."	◯	◯	◯	◯	◯
„Innovationsfonds-Projekte führen zu einer Verkomplizierung des Gesundheitswesens, z.B. weil neue Akteure oder Strukturen geschaffen werden."	◯	◯	◯	◯	◯
„Der Innovationsfonds bietet Hausärzt*innen die Chance, sich stärker in wissenschaftliche Forschung wie klinische Studien einzubringen."	◯	◯	◯	◯	◯
„Ich bezweifle, dass Innovationsfonds-Projekte am Ende den Weg in die Regelversorgung finden."	◯	◯	◯	◯	◯

10. Wie oft ist es bereits vorgekommen, dass Sie gefragt wurden, ob Sie an einem Innovationsfonds-Projekt teilnehmen möchten bzw. es Versuche gab, Sie für ein solches Projekt zu rekrutieren?

◯ Noch nie

◯ 1mal ◯ 2mal ◯ 3-5mal ◯ Mehr als 5mal ◯ Weiß nicht, keine Angabe

11. An wie vielen Innovationsfonds-Projekten haben Sie bereits teilgenommen oder tun dies aktuell? (Addieren Sie bitte frühere und aktuelle Teilnahmen.)

◯ An keinem => bitte direkt zu **Frage 23** übergehen

◯ An 1 ◯ An 2 ◯ An 3 ◯ An mehr als 3 ◯ Keine Angabe

12. Bezieht bzw. bezog sich Ihre Teilnahme auf alle oder nur auf bestimmte Krankenkassen?

◯ Auf alle Krankenkassen ◯ Nur auf bestimmte Krankenkassen ◯ Teils, teils

13. Welchen der folgenden Themengebiete lässt sich das/die Innovationsfonds-Projekt(e), an dem/denen Sie beteiligt sind bzw. waren, zuordnen? (Mehreres kann angegeben werden.)

◯	Versorgung in strukturschwachen bzw. ländlichen Gebieten
◯	Arzneimitteltherapie bzw. Arzneimitteltherapiesicherheit
◯	Telemedizin, Telematik, eHealth/mHealth
◯	Versorgung für spezielle Patient*innengruppen
◯	Delegation und Substitution von Leistungen
◯	Geriatrische Versorgung
◯	Kommunikation mit Patient*innen und Förderung der Gesundheitskompetenz
◯	Versorgung für Menschen mit Behinderung
◯	Sektorenübergreifende bzw. -unabhängige Versorgung
◯	Versorgung für spezifische Krankheiten/Krankheitsgruppen
◯	Stärkung der regionalen Gesundheitsversorgung
◯	Versorgung von vulnerablen Gruppen (z.B. pflegende Angehörige)
◯	Medizinische Leitlinien und deren (Weiter-)Entwicklung
◯	Sonstiges

14. Auf welchem Weg haben Sie zu dem/den Innovationsfonds-Projekt(en) gefunden?

◯ Ich habe mich selbst gemeldet (z.B. Reaktion auf Lesen einer Bekanntmachung/Werbung)

◯ Ich wurde aktiv von Projektmitarbeiter*innen oder Projektpartnern (z.B. Ärztenetzwerke, Krankenkassen) darauf angesprochen und habe mich infolgedessen zur Teilnahme bereit erklärt

◯ Anderer Weg

◯ Teils, teils

15. War es in Folge Ihrer Teilnahme erforderlich, Mitglieder Ihres Praxisteams zu schulen?

◯ Ja, war erforderlich ◯ Nein, war nicht erforderlich ◯ Teils, teils

16. Womit waren Sie hinsichtlich der Innovationsfonds-Projekte, an denen Sie bislang teilgenommen haben, zufrieden bzw. was ist Ihrer Einschätzung nach positiv hervorzuheben? Einige Stichpunkte genügen.

____

____

____

17. Und womit waren Sie hinsichtlich der Innovationsfonds-Projekte, an denen Sie bislang teilgenommen haben, eher unzufrieden bzw. was haben Sie als negativ erlebt?

____

____

____

18. Die Teilnahme an einem Innovationsfonds-Projekt kann dazu führen, dass Praxisroutinen verändert werden müssen, wodurch sich Komplikationen im Praxisablauf ergeben können. Gab es aufgrund Ihrer Teilnahme an einem oder mehreren Innovationsfonds-Projekten Einschränkungen bzw. Komplikationen im Praxisablauf? (Gemeint sind z.B. Verzögerungen oder Schwierigkeiten)

◯ Ja, starke Komplikationen ◯ Ja, moderate Komplikationen ◯ Nein, keine Komplikationen

19. Wie schätzen Sie das insgesamt ein: Wie stark hat die Versorgung bzw. Therapie der einbezogenen Patient*innen von den Innovationsfonds-Projekten, an denen Sie bislang teilgenommen haben, profitiert?

◯ Sehr stark ◯ Eher stark ◯ Eher nicht so stark ◯ Überhaupt nicht ◯ Schwer zu sagen

20. Wenn Sie versuchen würden, eine Bilanz zu ziehen: Inwiefern stand der (Mehr-)Aufwand aufgrund Ihrer Teilnahme an den Innovationsprojekten im Verhältnis zum Nutzen?

◯ Nutzen überwog deutlich ◯ Nutzen überwog etwas ◯ Nutzen und (Mehr-)Aufwand in etwa gleich

◯ (Mehr-)Aufwand überwog etwas ◯ (Mehr-)Aufwand überwog deutlich ◯ Schwer zu sagen

21. Ist es schon einmal vorgekommen, dass Sie Ihre Teilnahme an einem Innovationsfonds-Projekt vorzeitig, also vor Projektende, abgebrochen haben?

◯ Ja, schon vorgekommen ◯ Nein, nicht vorgekommen => bitte direkt zu **Frage 23** übergehen

22. Was war der Grund hierfür?

____

____

23. Welche Verbesserungen würden Sie sich für die Zukunft bei Innovationsfonds-Projekten wünschen?

____

____

____

24. Wie wichtig wäre es Ihrer Ansicht nach, wenn Hausärzt*innen stärker als bislang in die Konzeption und Entwicklung neuer Innovationsfonds-Projekte einbezogen werden?

◯ Sehr wichtig ◯ Eher wichtig ◯ Eher unwichtig ◯ Schwer zu sagen, weiß nicht

Zum Schluss bräuchten wir noch einige statistische Angaben von Ihnen. Wie auch der Rest des Fragebogens werden diese Informationen natürlich streng vertraulich behandelt und anonymisiert.

Sie sind…

◯ Männlich ◯ Weiblich ◯ Divers

Ihr **Alter**: _____

Wo befindet sich Ihre Praxis? In einer Gemeinde/Stadt mit …

◯ mehr als 100.000 ◯ 20.000 bis 100.000 ◯ 5.000 bis 20.000 ◯ unter 5.000 Einwohnern

Welches Niederlassungsmodell beschreibt Ihre Praxis am besten?

◯ Einzelpraxis (Sie sind der/die einzige Arzt/Ärztin) ◯ Einzelpraxis mit angestellten Ärzt*innen

◯ Gemeinschaftspraxis ◯ Medizinisches Versorgungszentrum ◯ Sonstiges

Wie viele Patient*innen behandelt Ihre Praxis im Quartal?

◯ 500 - 750 ◯ 751 - 1000 ◯ 1001 - 1500 ◯ 1501 - 2000 ◯ Mehr als 2000

Sind Sie Mitglied in einem **(regionalen) Ärzte- bzw. Praxisnetzwerk**?

◯ Ja ◯ Nein

Vielen Dank für Ihre Teilnahme!

Sollten Sie diese Befragung schriftlich ausgefüllt haben, nutzen Sie bitte den von uns beigefügten Rückumschlag.

Was möchten Sie uns noch mitteilen? Hier finden Sie Platz für Anregungen, Kommentare und Kritik.

____

____

____
